# Prognostic Value of an m6A RNA Methylation Regulator-Based Signature in Patients with Hepatocellular Carcinoma

**DOI:** 10.1155/2020/2053902

**Published:** 2020-07-15

**Authors:** Xiaomin Wu, Xiaojing Zhang, Leilei Tao, Xichao Dai, Ping Chen

**Affiliations:** ^1^Department of Oncology, Yancheng No. 1 People's Hospital, Nantong University, Yancheng 224200, China; ^2^Department of Gynecologic Oncology, Zhejiang Cancer Hospital, Hangzhou 315000, China

## Abstract

**Purposes:**

Hepatocellular carcinoma (HCC) is one of the most common malignant tumors in the world. Recent researches have demonstrated that m6A methylation regulators play a key role in various cancers, such as gastric cancer and colon adenocarcinoma. Several m6A methylation regulators are reported to predict the prognosis of HCC. Therefore, there is a need to further identify the predictive value of m6A methylation regulators in HCC.

**Methods:**

We utilized The Cancer Genome Atlas (TCGA) database to obtain the gene expression profile of m6A RNA methylation regulators and clinical information for patients with HCC. Besides, we identified two clusters of HCC with various clinical factors by consensus clustering analysis. Then the least absolute shrinkage and selection operator (LASSO) and the Cox regression analysis were applied to construct a prognostic signature.

**Results:**

Except for ZC3H13 and METTL14, a majority of the thirteen m6A RNA methylation regulators were significantly overexpressed in HCC specimens. HCC patients were classified into two groups (cluster 1 and cluster 2). The cluster 1 was with a significantly worse prognosis than cluster 2, and most of the 13 known m6A RNA methylation regulators were upregulated in cluster 1. Besides, we developed a prognostic signature consisting of YTHDF2, YTHDF1, METTL3, KIAA1429, and ZC3H13, which could successfully differentiate high-risk patients. More importantly, univariate and multivariate Cox regression analysis indicated that the signature-based risk score was an independent prognostic factor for patients with HCC.

**Conclusions:**

Our study showed these five m6A RNA methylation regulators can be used as practical and reliable prognostic tools of HCC, which might have potential value for therapeutic strategies.

## 1. Introduction

Hepatocellular carcinoma (HCC), accounting for up to 90% of all primary liver cancers, is the 4th most common cause of death worldwide in 2018. The incidence and mortality of HCC continue to increase in almost all countries [1]. Clinically, some curative treatment strategies, such as surgical resection and liver transplantation, are feasible for most early stage patients [2, 3]. Unfortunately, most patients with HCC are in an advanced stage of the disease at the time of diagnosis. Although target therapy and immune checkpoint inhibitors have been proven to improve survival in metastatic patients in recent years, the median survival is still less than two years [4, 5]. Therefore, exploring novel biomarkers and therapeutic targets for HCC diagnosis and treatment is still a challenging issue.

N6-methyladenosine (m6A) is the most common internal modification found in messenger RNAs (mRNAs), microRNAs, and long noncoding RNAs, where it plays critical regulatory roles in transcription, processing, and metabolism [6]. Several enzymes responsible for m6A RNA modifications, which are composed of methyltransferases (writers), binding proteins (readers), and demethylases (erasers), have been identified. METTL3, METTL14, KIAA1429, WTAP, RBM15, and ZC3H13 are involved in the methylated modification of RNA, while FTO and ALKBH5 could exhibit efficient m6A demethylase activity. Expect for HNRNPC, all known m6A readers are members of the YTH domain-containing family that include YTHDF and YTHDC subtypes [7].

Recent evidence has proved that the expression of m6A modulators is closely tied to distinct pathological processes, including stem cell differentiation, tumorigenicity, and metastasis [8]. Some findings suggest that m6A RNA methylation regulators serve as potential clinical molecular markers, thereby offering fresh insights into the treatment of cancers, such as cervical cancer, pancreatic cancer, hepatocellular carcinoma, and acute myeloid leukemia [9–12]. Although METTL3 is upregulated in HCC and acts as an oncogene in HCC [13], we have little knowledge of the relationship between m6A-related regulators and HCC. Therefore, there is a need to further identify the prognostic significance of m6A methylation regulators in HCC.

In our article, we analyzed gene expression characteristics and evaluated associations between m6A methylation regulators and clinical and pathological features in 374 patients with HCC. Five candidate genes were identified from 13 m6A-related genes, which may be independent prognostic biomarkers.

## 2. Methods

### 2.1. Datasets

We obtained the RNA-Seq transcriptome data and relevant clinical data for patients with primary HCC from The Cancer Genome Atlas (TCGA, https://cancergenome.nih.gov/). All RNA-Seq gene expression data have been normalized by using Perl. A total of 374 HCC cases and 50 normal control samples were included for subsequent analysis.

### 2.2. Differentially Expressed m6A RNA Methylation Regulator in HCC

There are thirteen m6A RNA methylation regulators, including YTHDC1, YTHDC2, ZC3H13, METTL3, METTL14, YTHDF1, YTHDF2, KIAA1429, ALKBH5, FTO, WTAP, HNRNPC, and RBM15. We identified those differentially expressed regulators between HCC samples and standard control samples by using the “EdgeR” package.

### 2.3. Consensus Clustering Analysis

We removed 50 normal cases and grouped 374 cancer cases by clustering the cases based on consensus expression of m6A RNA methylation regulators with the ConsensusClusterPlus package [14]. Principal component analysis (PCA) was employed to verify the results of the cluster by the limma package [15].

### 2.4. Risk Characteristics and Prognosis

To evaluate the association between 13-gene expression and patients' survival, Cox regression analysis and least absolute shrinkage and selection operator (LASSO) were performed. After independent m6A regulatory genes were identified and their coefficients were determined, we used m6A regulatory gene-based risk score prediction model to stratify HCC patients into high-risk and low-risk groups. The receiver operating characteristic (ROC) curve was used to detect the predictive efficiency of the survival model [16]. The valuable influence factors (including age, sex, histologic grade, and pathologic stage) of HCC patients were filtrated and formulated with a multivariate Cox regression analysis model.

### 2.5. Statistical Analysis

All statistical data were analyzed by using R version 3.6.1 (https://www.r-project.org/). All statistical tests were 2-sided, and *p* values < 0.05 were considered statistically significant. If some information is missing, we will delete the associate sample from the analysis. One-way ANOVA compared the expression of 13 regulators in HCC and normal tissues in TCGA samples. The association between clinical characteristics and m6A RNA methylation regulatory genes was analyzed with the chi-square test. The Kaplan–Meier curve with a log-rank test was adopted to compare the survival outcome.

## 3. Results

### 3.1. Profiling of m6A RNA Methylation Regulators in HCC

**Figure 1:**
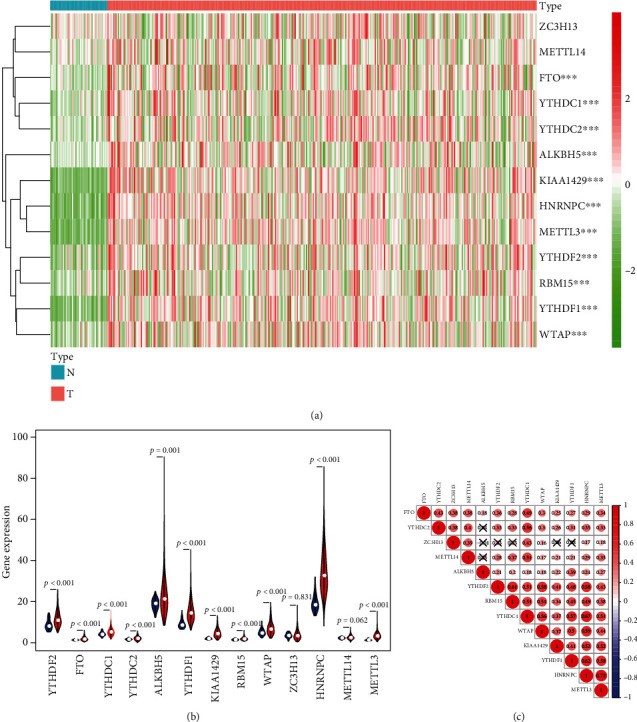
The profiling of m6A RNA methylation regulators in hepatocellular carcinoma. (a) The heatmaps of 13 m6A RNA methylation regulators in tumor tissues and normal tissues (red is upregulated and green is downregulated; ∗*p* < 0.05, ∗∗*p* < 0.01, and ∗∗∗*p* < 0.001). (b) Vioplot visualizing the differentially m6A RNA methylation regulators in hepatocellular carcinoma (red is gastric cancer and blue is normal). (c) Spearman's correlation analysis of the 13 m6A modification regulators in hepatocellular carcinoma.

Heatmap and violin plot were presented to summarize the specially expressed m6A RNA methylation regulators between HCC samples and control samples (Figures 1(a) and 1(b)). Most increased regulators in tumor cases compared to normal cases were YTHDF2 (*p* < 0.001), FTO (*p* < 0.001), YTHDC1 (*p* < 0.001), YTHDC2 (*p* < 0.001), YTHDF1 (*p* < 0.001), KIAA1429 (*p* < 0.001), METTL3 (*p* < 0.010), WTAP (*p* < 0.001), RBM15 (*p* < 0.001), HNRNPC (*p* < 0.001), and ALKBH5 (*p* = 0.001). ZC3H13 (*p* = 0.831) and METTL14 (*p* = 0.062) showed no significant difference. We also performed the correlation analysis among the 13 m6A RNA methylation regulators and found that HNRNPC and METTL3 were most relevant. FTO, YTHDC1, WTAP, HNRNPC, and METTL3 were correlated with all the other 12 genes, respectively (Figure 1(c)).

### 3.2. Consensus Clustering of 13 m6A RNA Methylation Regulators

**Figure 2:**
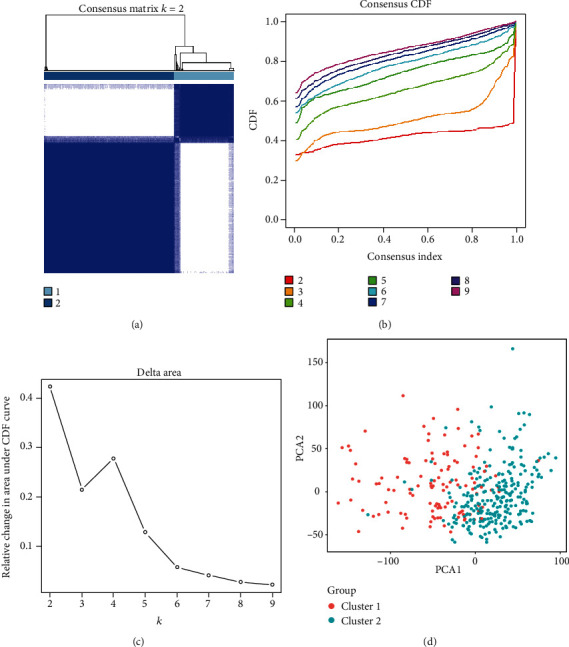
Identification of consensus clusters by m6A RNA methylation regulators. (a) Consensus clustering matrix for *k* = 2. (b) Consensus clustering cumulative distribution function (CDF) for *k* = 2 to 9. (c) Relative change in area under CDF curve for *k* = 2 to 9. (d) Principal component analysis of the total RNA expression profile in the TCGA dataset. Hepatocellular carcinoma in the cluster 1 subgroup is marked with orange, and the cluster 2 subgroup is marked with green.

Due to small numbers in one of these clusters, we did not divide HCC samples into three groups. Then, we clustered 374 HCC samples into two groups (termed as cluster 1 and cluster 2, respectively) based on the expression of m6A RNA methylation regulators in TCGA (Figures 2(a)–2(c)). We further proved the correctness about our grouping by PCA. Significant variance between the two subgroups is shown in the PCA modal (Figure 2(d)).

**Figure 3:**
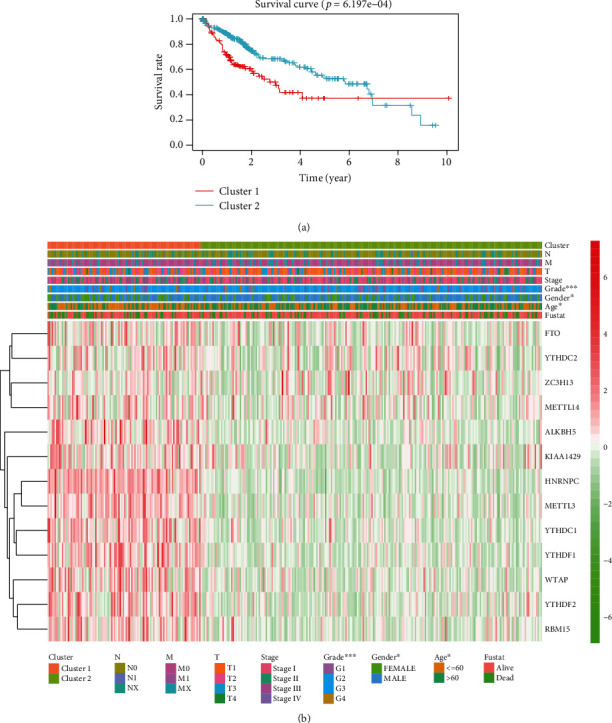
Differential clinicopathological features and overall survival of hepatocellular carcinoma in the cluster 1/2 subgroups. (a) The Kaplan–Meier overall survival (OS) curves for 374 TCGA hepatocellular carcinoma patients. Gastric cancer patients in the cluster 1 subgroup are marked with red, and those in the cluster 2 subgroup are marked with blue. (b) Heatmap and clinicopathologic features of the two clusters defined by the m6A RNA methylation regulators consensus expression (red is upregulated and green is downregulated; ∗*p* < 0.05, ∗∗*p* < 0.01, and ∗∗∗*p* < 0.001).

Notably, we also observed that HCC patients in cluster 1 were with an obviously worse prognosis than cluster 2 (Figure 3(a), *p* value = 6.197e − 04). In addition, we discovered that most of m6A RNA methylation regulators were significantly expressed in the cluster 1 group. In HCC, some clinical characteristics, such as tumor stage, tumor topography, lymph nodal, and metastasis, did not vary significantly between two subgroups. However, compared with the cluster 2 group, the cluster 1 group was strongly associated with females, higher grade, and younger age (Figure 3(b)).

### 3.3. Construction and Validation of the Five Prognostic Signatures

**Figure 4:**
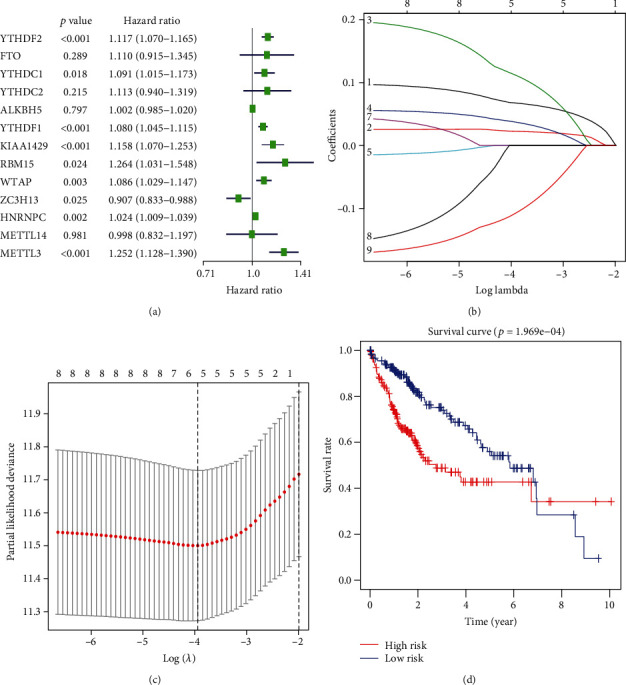
The effect of m6A RNA methylation regulators on the prognosis of hepatocellular carcinoma. (a) Cox univariate analysis of m6A RNA methylation regulators. (b, c) The coefficients calculated by multivariate Cox regression using LASSO are shown. (d) The Kaplan–Meier overall survival curves for patients in the TCGA datasets assigned to high- and low-risk groups based on the risk score.

**Figure 5:**
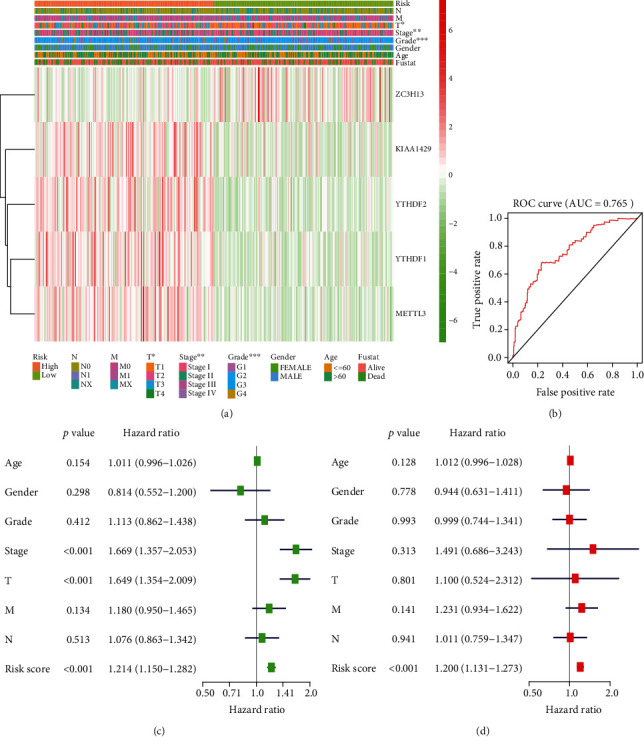
Effects of the risk score and clinicopathological variables on the prognosis of hepatocellular carcinoma patients. (a) The heatmap shows the expression of five m6A RNA methylation regulators and the distribution of clinicopathological variables between the high- and low-risk groups (red is upregulated and green is downregulated; ∗*p* < 0.05, ∗∗*p* < 0.01, and ∗∗∗*p* < 0.001). (b) ROC curves showed the predictive efficiency of the risk signature. (c) Cox univariate analyses of clinicopathological variables (including the risk score) and overall survival. (d) Cox multivariate analyses of clinicopathological variables (including the risk score) and overall survival.

The expression of m6A RNA methylation regulators was exposed to univariate COX regression, and nine genes (YTHDF2, YTHDF1, METTL3, KIAA1429, HNRNPC, WTAP, YTHDC1, RBM15, and ZC3H13) related to the overall survival (OS) were measured as predictive genes (*p* < 0.05) for LASSO analysis. Finally, these five regulators were selected to construct a risk signature, and the coefficients were extracted from the LASSO algorithm (Figures 4(a)–4(c)). The risk score formula to predict OS was developed as follows: risk score = (0.068∗YTHDF2) + (0.023∗YTHDF1) + (0.113∗METTL3) + (0.038∗KIAA1429) + (−0.109∗ZC3H13). Therefore, patients were separated into high-risk group (*n* = 182) and low-risk group (*n* = 183) based on the median risk score. The distributions of risk scores and patients' survival time and status are shown in Figure 4(d). The survival analysis presented that patients in the high-risk group generally had poorer OS than those in the low-risk group (*p* = 1.969e − 04). We also assessed the prognostic value of these five risk genes using time-dependent ROC analysis. The results demonstrated that the area under the ROC curve (AUC) for 0.5-, 1-, 3-, and 5-year OS was 0.731, 0.765, 0.723, and 0.619, respectively (Figure 5(b), AUC for 1-year OS).  Figure 5(a) shows the expression of five prognostic genes between the high-risk set and low-risk set. For instance, four risk genes (KIAA1429, YTHDF2, YTHDF1, and METTL3) were highly expressed, and the protective ZC3H13 was lowly expressed in the high-risk set. Moreover, we found significant differences of five selected m6A RNA methylation regulators between two groups with respect to T status (*p* < 0.05), stage (*p* < 0.01), and grade (*p* < 0.001).

To investigate whether the prognostic signature-based risk score was an independent negative prognostic indicator, the univariable and multivariable Cox regression analyses were used in 231 samples containing the complete data. The univariate Cox regression showed that stage, T status, metastasis, and risk score (stage: *p* < 0.001; T status: *p* < 0.001; metastasis: *p* = 0.023; risk score: *p* < 0.001; Figure 5(c)) were predictors for OS. Moreover, multivariate Cox regression analysis further identified that risk score (HR = 1.193, 95%CI = 1.111 − 1.281, *p* < 0.001; Figure 5(d)) was the only significant independent risk factor.

**Figure 6:**
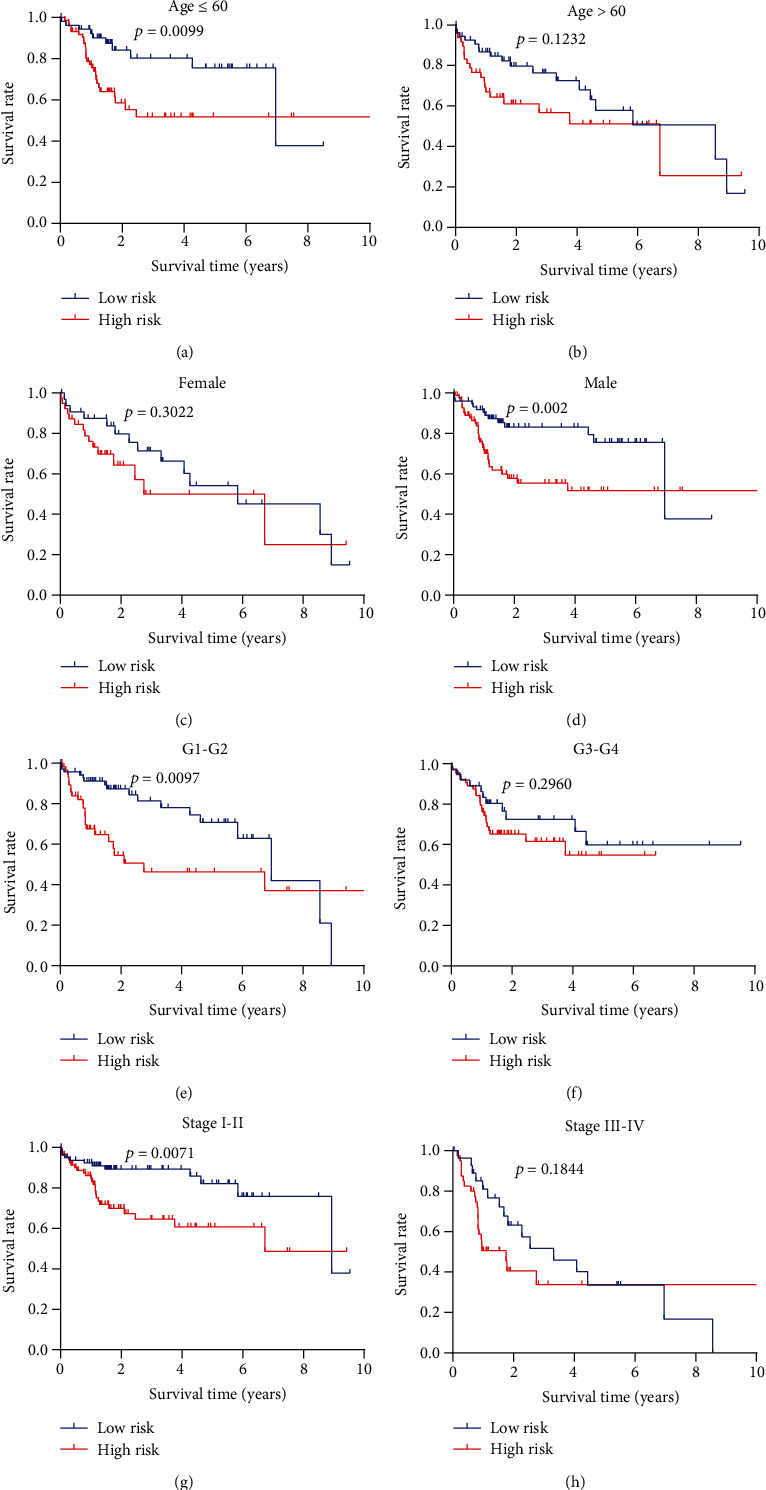
Prognostic value of the risk signature in hepatocellular carcinoma patients classified into specific cohorts. The Kaplan–Meier survival curve for patients with (a) age < = 60, (b) age > 60, (c) female, (d) male, (e) grade 1-grade 2, (f) grade 3-grade 4, (g) stages I-II, (h) stages III-IV.

The risk signature for different clinicopathological variables (age, gender, grade, and stage) also had the merits of prognosis (Figures 6(a)–6(h)). These results suggested that patients in the low-risk set had an obviously longer OS than those in the high-risk set for those with age ≤ 60 (*p* = 0.0099), male cases (*p* = 0.002), G1-G2 (*p* = 0.0097), or stages I-II (*p* = 0.0071). However, there was no significant difference between these two groups in terms of age > 60 (*p* = 0.1232), female cases (*p* = 0.3022), G3-G4 (*p* = 0.2906), and stages III-IV (*p* = 0.1844).

## 4. Discussion

Epidemiological and clinical evidence suggests that hepatocellular carcinoma is caused by the interaction of viruses, environmental factors, and genetic predisposition [17]. Except for the epigenetic changes on DNA, RNA modification has also attracted much attention from researchers recently. There are over 100 known RNA modifications identified by high-throughput sequencing [18]. The most abundant RNA modification, N6-methyladenosine (m6A), has been found in human diseases, including infection, type II diabetes, heart disease, and cancer [19–22]. M6A RNA methylation regulator was observed to be tightly related to carcinogenesis and poor prognosis in patients with HCC [23]. In our research, we compared the expression of thirteen m6A RNA methylation regulators in HCC and control samples using data from TCGA and found that the majority of these regulators were upregulated in tumor samples. Besides, the HCC cases were separated into two clusters with significant differences for OS and clinical features. Then, remarkable differences in OS were found between high-risk and low-risk groups. Finally, a risk signature including five m6A RNA methylation regulators could serve as a prognostic factor for patients with HCC.

In the present study, it should be noted that all m6A RNA methylation regulators were upregulated in cancerous tissues, and the expression of almost all regulators increased most significantly except ZC3H13 and METTL14. These results may imply that dysregulation of m6A might play an important role in HCC tumorigenesis. Recent researches have shown that some regulators play essential and diverse biological functions in the genesis and development of various cancers [24]. Similar to HCC, Cai et al. conclude that METTL3 was overexpressed in breast cancer and colorectal cancer compared to normal control and might behave as an oncogene in tumorigenesis [25, 26]. Surprisingly, a study by Deng et al. found that METTL3 is not only lowly expressed in colorectal cancer, but its high expression was also correlated with longer survival time [27]. METTL3 was also observed to be significantly decreased in endometrial cancer tissues compared to the adjacent normal tissues [28]. It was known that the catalytic activity of m6A “writer” and additional “reader” was essential, and m6A modification relied on reader proteins to elicit a variety of biological functions. Chen et al. demonstrate that METTL3 epigenetically silenced suppressor of cytokine signaling 2 in HCC through an m6A-YTHDF2-dependent mechanism [13]. Interestingly, YTHDF2 has dual functions in pancreatic cancer by promoting proliferation and restraining migration and invasion [29]. These controversial findings could provide different insights into the underlying mechanism of related regulators in the progression of human malignancies, even in the same cancer, and more work remains to be done in this field.

Our study indicated that all five final regulators were correlated with the prognosis of HCC patients. COX regression analysis for risk score and clinical features confirmed that YTHDF2, YTHDF1, METTL3, KIAA1429, and ZC3H13 could serve as markers to predict prognosis and may even be targeted in novel therapies of HCC. KIAA1429 was demonstrated to be correlated with the proliferation and metastasis of the hepatocellular carcinoma. After the disjunction of HuR and the downregulation of GATA3 pre-mRNA, KIAA1429 mediates m6A methylation on the 3′ UTR of GATA3 pre-mRNA in liver cancer cells guided by GATA3-AS [30]. Lan et al. revealed that KIAA1429 was a redoubtable driver of liver cancer development and metastasis, which showed that KIAA1429 could be used as a novel gene for treating HCC patients.

Our results suggested that ZC3H13 had no significant difference between HCC cases and normal cases. Furthermore, by comparing the expression of m6A RNA regulators in cluster 1 and 2 subgroups, the difference of ZC3H13 expression between the two subgroups was not significant. Interestingly, our prognostic model demonstrated that the expression level of ZC3H13 was positively correlated with the prognosis of HCC, suggesting that it might function as a tumor suppressor in HCC. It was reported that the downregulation of ZC3H13 expression regulated KRAS and ERK signaling expression, which could inhibit the invasion and proliferation of colorectal cancer cells [31]. More and more evidence suggested that a variety of growth factors promote HCC cell multiplication through the activation of the Ras/Raf-1/ERK pathway, and the RAS-ERK pathway plays a crucial role in the tumorigenesis of HCC [32, 33]. However, the role of ZC3H13 and the RAS-ERK pathway in the molecular mechanism of HCC has not been elucidated yet. Future studies could focus on their relationship to develop relevant therapeutics for the treatment of HCC.

However, there are also some potential limitations in the current study. First, the mainly American patients were downloaded from TCGA, which might lead to selection bias. Second, the number of HCC cases was much more than that of normal cases. It might have great influence on the reliability and accuracy of the results. Third, some clinical parameters, such as alcohol assumption and the level of hepatitis virus DNA, were not taken into consideration. Finally, future studies in different populations and databases are required to validate our results.

## 5. Conclusions

In summary, we found that the expression of m6A RNA methylation regulators was tightly linked to the prognosis of HCC. Our study had the crucial probative value to prove the role of m6A RNA methylation in HCC. Meanwhile, YTHDF2, YTHDF1, METTL3, KIAA1429, and ZC3H13 might be a potential predictor and therapeutic target for HCC. Further biochemical studies and functional experiments are required to confirm these results in the future.

## Data Availability

The data used to support the findings of this study are available from the corresponding author upon request.
